# Supplementary material for: The genome sequence of the Dotted Ermel moth, Ethmia dodecea (Haworth, 1828)
Source: Wellcome Open Res. 2025 Feb 24;10:93. [Version 1] doi: 10.12688/wellcomeopenres.23750.1 (PMC12209772; doi:10.12688/wellcomeopenres.23750.1)
Supplement: Peer review history

# Reviewer report: Reviewer response for version 1

**DOI:** 10.21956/wellcomeopenres.26198.r123250

**Published:** 2025-06-30

**Authors:** Annabel Whibley

**Affiliations:** 1 Grapevine Improvement, Bragato Research Institute, Lincoln, Lincoln, New Zealand

Boyes and colleagues report the genome assembly of the Dotted Ermel Moth ( *Ethmia dodecea). *No genome annotation is provided in this report. The quality metrics of this assembly are superb and required relatively scant curation intervention.  99.9% of the assembly has been scaffolded into 29 pseudochromosomes with excellent QV and completeness. Specimen ID has been validated by barcode sequencing.  The Data Note follows Darwin Tree of Life project protocols, assembly and annotation pipelines and reporting templates. Appropriate methods are used, metadata is comprehensive and public accession links are functional. This promises to be a wonderful genomic resource for the community.

Are sufficient details of methods and materials provided to allow replication by others?

Yes

Is the rationale for creating the dataset(s) clearly described?

Yes

Are the datasets clearly presented in a useable and accessible format?

Yes

Are the protocols appropriate and is the work technically sound?

Yes

Reviewer Expertise:

Genomics, Evolution, Bioinformatics

I confirm that I have read this submission and believe that I have an appropriate level of expertise to confirm that it is of an acceptable scientific standard.

# Reviewer report: Reviewer response for version 1

**DOI:** 10.21956/wellcomeopenres.26198.r123252

**Published:** 2025-05-19

**Authors:** Arun Arumugaperumal

**Affiliations:** 1 Rajalakshmi Engineering College, Chennai, Tamil Nadu, India

The data note descibes the whole genome sequencing project of a moth *Ethmia dodecea*. The genome assembly presented was of size 457.55 Mb. The assembly sequence was arranged into 29 chromosome molecules. The mitochondria genome was reported of size 15.34 kb. This is the first report of the genome sequence of the dotted Ermel moth. 

 The coverage based on sequencing data was reported as 152x. But this might be calculated by using the genome size obtained from GenomeScope which is 453.39 Mb. In this report the final assembly was 457.55 Mb and the assembly also had underwent decontamination through ASCC pipeline. So the coverqage value need to be racalculated.

 BUSCO analysis shows that the genome assembly was 98.5% complete. The contig N50 was reported as  12.42 Mb. This indicates the good quality of the genome. The accession numbers mentioned in the data note were all working fine.

Are sufficient details of methods and materials provided to allow replication by others?

Yes

Is the rationale for creating the dataset(s) clearly described?

Yes

Are the datasets clearly presented in a useable and accessible format?

Yes

Are the protocols appropriate and is the work technically sound?

Partly

Reviewer Expertise:

Bioinformatics, Genomics

I confirm that I have read this submission and believe that I have an appropriate level of expertise to confirm that it is of an acceptable scientific standard, however I have significant reservations, as outlined above.

# Reviewer report: Reviewer response for version 1

**DOI:** 10.21956/wellcomeopenres.26198.r123248

**Published:** 2025-05-19

**Authors:** Kay Lucek

**Affiliations:** 1 University of Neuchâtel, Neuchâtel, Switzerland

The authors present the chromosome level genome assembly of a male specimen of the Dotted Ermel moth Ethmia dodecea. The assembly consists of 29 chromosomes and is not annotated for genes. The assembly is highly complete as revealed by the high BUSCO score but not fully phased. The assembly has been generated using the established pipelines of the Darwin Tree of Life Consortium.

 The presented assembly will be of great value to study the evolution of Lepidoptera. Surprisingly there is no information about the biology of the species or its distribution in the UK and beyond. If such information is missing, it would be nice to have it highlighted.

 According to Wikipedia, the species was initially described in England and is found in Europe, Asia Minor, Iran and Siberia. Larvae feed on Lithospermum officinale.

Are sufficient details of methods and materials provided to allow replication by others?

Yes

Is the rationale for creating the dataset(s) clearly described?

Yes

Are the datasets clearly presented in a useable and accessible format?

Yes

Are the protocols appropriate and is the work technically sound?

Yes

Reviewer Expertise:

Speciation Genomics

I confirm that I have read this submission and believe that I have an appropriate level of expertise to confirm that it is of an acceptable scientific standard.
